# The content and delivery of psychological interventions for perinatal depression by non-specialist health workers in low and middle income countries: A systematic review^☆^

**DOI:** 10.1016/j.bpobgyn.2013.08.013

**Published:** 2014-01

**Authors:** Neerja Chowdhary, Siham Sikander, Najia Atif, Neha Singh, Ikhlaq Ahmad, Daniela C. Fuhr, Atif Rahman, Vikram Patel

**Affiliations:** a Sangath, 841/1, Alto Porvorim – Goa 403511, India; b The London School of Hygiene and Tropical Medicine, UK; c Human Development Research Foundation, Islamabad, Pakistan; ^d^University of Liverpool, Institute of Psychology, Health and Society Child Mental Health Unit, Alder Hey Children's NHS Trust, Mulberry House, Eaton Road, Liverpool L12 2AP, UK

**Keywords:** perinatal depression, depression, mothers, allied health personnel, community health workers, voluntary workers, developing country

## Abstract

Psychological interventions delivered by non-specialist health workers are effective for the treatment of perinatal depression in low- and middle-income countries. In this systematic review, we describe the content and delivery of such interventions. Nine studies were identified. The interventions shared a number of key features, such as delivery provided within the context of routine maternal and child health care beginning in the antenatal period and extending postnatally; focus of the intervention beyond the mother to include the child and involving other family members; and attention to social problems and a focus on empowerment of women. All the interventions were adapted for contextual and cultural relevance; for example, in domains of language, metaphors and content. Although the competence and quality of non-specialist health workers delivered interventions was expected to be achieved through structured training and ongoing supervision, empirical evaluations of these were scarce. Scalability of these interventions also remains a challenge and needs further attention.

## Introduction

Perinatal depression is defined as an episode of depression occurring either during pregnancy, within 1 year after delivery, or both [1], [2]. Maternal depression is a more loosely defined term that includes perinatal depression but also depression in mothers with young children. We use the term perinatal depression in this broader context. Systematic reviews conducted in high-income countries have shown that about 10% of pregnant women and 13% of those who have given birth experience depression (or anxiety, which frequently co-occurs with depression). The prevalence of perinatal depression is higher in low- and middle-income countries (LMIC), with a mean prevalence of 15.6% (95% CI 15.4 to 15.9) antenally and 19.8% (95% CI 19.5 to 20.0) postnatally, particularly in poorer women with gender-based risks (including intimate partner violence, the bias against female babies, and role restrictions regarding housework and infant care) or a psychiatric history [3].

The combination of this high prevalence of perinatal depression in LMIC as well as the woman's primary responsibility for childcare, means that, apart from its effect on maternal health, perinatal depression can have a substantial influence on child health outcomes [4], [5], [6], [7], [8], [9]. A recent systematic review of studies from LMIC reported that children of mothers with depression or depressive symptoms are more likely to be underweight (OR 1.5; 95% CI 1.2 to1.8) or stunted (OR 1.4; 95% CI 1.2 to1.7) [10]; the review estimated that between 23 and 29% fewer children would be underweight or stunted if the infant population were entirely unexposed to perinatal depressive symptoms. Perinatal depression has hence been described as a global threat to child development [11], and is recognised as a major public health concern, especially in LMIC [4], [12].

In high income countries, evidence shows that psychosocial and psychological interventions compared with usual postpartum care are effective in reducing perinatal depression [13]. Few LMIC have sufficient mental health professionals available to meet the population's needs [14]. Considering the limited availability of specialised resources, it is necessary to explore alternative delivery strategies in LMIC [15]. Task-shifting or task-sharing to non-specialist health workers (NSHW) is emerging as an effective way to improve the access to health services and specifically services for mental disorders [16]. These include healthcare practitioners (e.g. doctors, nurses, community health workers) and non-professionals (e.g. lay providers) [17]. A recently conducted meta-analysis of perinatal depression interventions in LMIC included 13 (published as well as unpublished) trials with 20,092 participants [18]. In all but one of these studies, the interventions were delivered by NSHW. Mothers and children benefitted significantly from the interventions tested compared with routine care (pooled effect size 0.38, 95% CI −0.56 to −0.21). Where assessed, benefits to the child included improved mother–infant interaction, better cognitive development, reduced diarrhoeal episodes, and increased rates of immunisation.

The aim of this review is to describe the content and delivery of such interventions. We sought to specifically address the following questions: (1) What are the types of interventions for perinatal depression in LMIC delivered by NSHW?; (2) What are the psychosocial strategies and techniques that the interventions utilise?; (3) What are the adaptations required to make these interventions culturally and contextually appropriate?; (4) What are the adaptations required to make these interventions deliverable by NSHW?; (5) What are the characteristics of the NSHWs, their training and supervision?; and (6) What are the challenges encountered in intervention delivery and how were these addressed?

## Method

Studies were identified by a systematic literature search using the following strategies: (1) a database search of Ovid Medline, EMBASE and PsycINFO until December 31, 2012, was conducted to identify studies from LMIC describing interventions for perinatal depression delivered by NSHW. Search terms were adapted from another systematic review [17] and have been listed in Appendix 1. No start date was specified; and (2) cross-referencing of eligible articles to identify additional studies that met our inclusion criteria.

### Inclusion criteria

Criteria for inclusion consisted of psychological treatments for perinatal depression in LMIC (according to the World Bank classification, July 2012) delivered by any type of NSHW. Studies involving women with perinatal depression, defined as a non-psychotic depressive episode or the presence of depressive symptoms that begins during pregnancy or in the early postnatal period (within 6 weeks of delivery) were included.

### Exclusion criteria

Studies conducted with women with psychotic depression, depressive episode in a woman with bipolar disorder or other co-morbidities were excluded; studies on interventions involving provision by specialists (i.e psychiatrists, psychologists, psychiatric nurses, mental health social workers), and also studies conducted in high-income countries were excluded.

### Data extraction

The titles and abstracts of each citation identified from the search were independently inspected by two reviewers (NC, NA) with reference to the inclusion and exclusion criteria. The potentially relevant full-text papers were accessed and independently reviewed by the two reviewers. Any disagreements were resolved by consensus and, when this could not be reached, a third reviewer (VP) adjudicated. Papers that referenced previous publications describing the details of the interventions and adaptations made were also retrieved. Data were summarised in a table based on the research questions identified for the review.

### Data analysis

Thematic analysis was used to evaluate the strategies used in interventions, NSHW features and challenges encountered in intervention delivery. We followed the process of distillation [19], which is a method whereby interventions are conceptualised not as single units of analysis, but rather as composites of individual strategies, techniques, or components that can allow subsequent empirical grouping. Bernal's framework [20] was used for analysis of the nature of the cultural adaptations. The framework comprises eight dimensions that can be the targets of cultural adaptations: (1) language of the intervention; (2) therapist matching; (3) cultural symbols and sayings (metaphors); (4) cultural knowledge or content; (5) treatment conceptualisation; (6) treatment goals; (7) treatment methods; and (8) treatment context. Analysis was both deductive, consisting of pre-determined categories applied to data, and inductive (i.e. inferring themes from the coded data).

## Findings

### Description of the studies

**Fig. 1:**
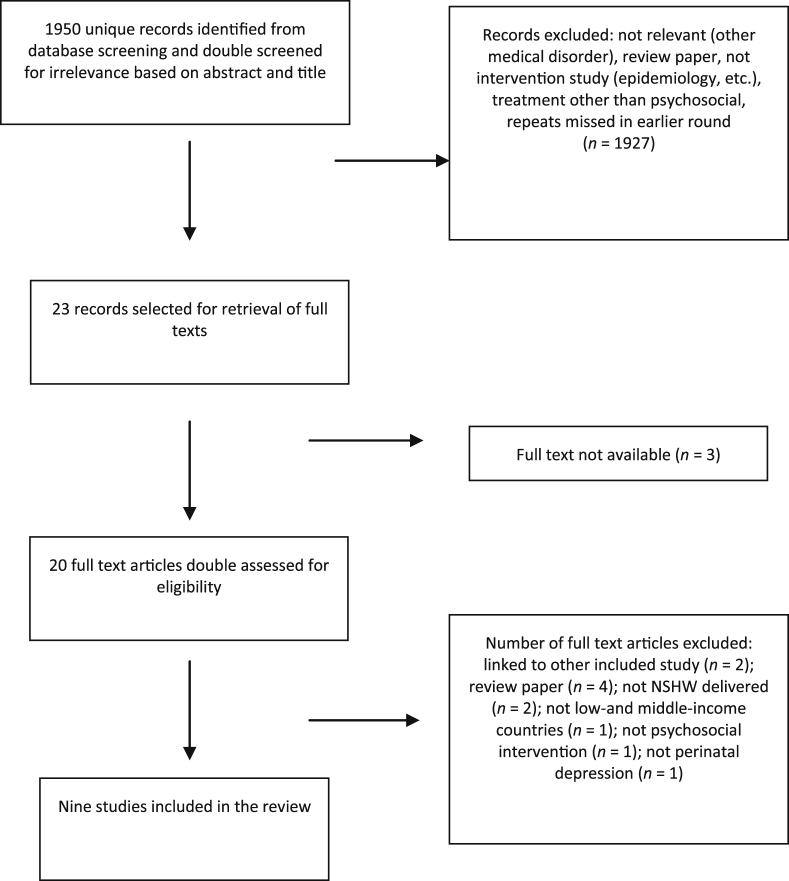
Studies included in the review. NSHW, non-specialist health workers.

After removing duplicates, the electronic search identified 1950 potential studies. The flow chart of studies from this starting point is shown in Fig. 1.

Nine studies were selected for final inclusion in this review ∗[21], ∗[22], ∗[23], ∗[24], ∗[25], ∗[26], ∗[27], ∗[28], ∗[29] The characteristics of the included studies are described in Table 1. All the studies were written in the English language. Two studies were conducted in South Africa ∗[24], [30], two in Chile ∗[21], ∗[26], and one each from China [25], Jamaica [22], India [29], Pakistan [27] and Turkey [28]. One study used a pilot non-randomised-controlled study design [24], one used a pre-test–post-test semi-experimental model [28], whereas the other seven studies were randomised-controlled trials. Of these, three studies used a cluster randomised-controlled design ∗[22], ∗[27], ∗[29], whereas the remaining used individual level randomisation ∗[21], ∗[25], ∗[26], [30]. Although studies measured perinatal depressive symptoms as an outcome, in four studies this was the primary outcome whereas, in the remaining studies, the primary outcomes were the physical health of mother and infant, quality of mother– child interaction, infant weight and height, child development and HIV knowledge.

**Table 1:** Characteristics of the nine studies included in the review of perinatal depression interventions provided by non-specialist health workers in low- and middle-income countries.

Author	Location	Design	Sample	Comparison group	Primary outcome	Secondary outcome	Result (Outcome - maternal depression)
Aracena M, 2009 [21]	Chile	Experimental RCT	Adolescent mothers (14–19 years), first pregnancy. Intervention group *n* = 45; control group; *n* = 45	Standard prenatal and well baby care at health centres	Physical health of mother and infant	Maternal mental health using the GHQ at the end of the intervention	Intervention group: average 10.94 points (SD: 5.58). Control group: average 13.85 (SD: 6.99). t (89) = 2.20; *P* = 0.031.
Baker-Henningham, 2005 [22]	Jamaica	Cluster RCT	Mothers of under-nourished children aged 9–30 months attending 18 nutrition clinics. Intervention group *n* = 64; control group *n* = 61	Standard health and nutrition care	Child development	Maternal depression using CES-D at end of 1 year	Effect size b = −0.98; 95% CI −1.53 to −0.41). The change was equivalent to 0.43 SD. Mothers receiving more than 40 visits and mothers receiving 25–39 visits benefited significantly from the intervention (b = −1.84, 95% CI −2.97 to −0.72, and b = −1.06; 95% CI −2.02 to −0.11, respectively), whereas mothers receiving less than 25 visits did not benefit.
Cooper PJ, 2009 [23]	South Africa	Individual RCT	Women in the last trimester of pregnancy. Intervention group *n* = 220; control group *n* = 229.	Standard care provided by local infant clinic	Quality of mother–infant interactions at 6 and 12 months postpartum; infant attachment security at 18 months	Maternal depression (a dichotomous variable for depressive disorder using SCID, and a continuous variable for depressive symptoms using EPDS) assessed at 6 and 12 months.	At 6 months effect size = 2.05; *P* = 0.041; At 12 months effect size = 0.24, *P* = 0.813 .
Futterman D, 2013 [24]	South Africa	Pilot non-randomised- controlled trial	Pregnant women attending maternity clinics who were HIV positive; 160 enrolled. Number followed up: intervention group *n* = 40; control group *n* = 31.	Standard PMTCT care	HIV knowledge, discomfort. Social support, satisfaction	Depression using the CES-D; 6 months after intervention.	Depression scores reduced significantly more in the intervention than in the control group (14.0 to 5.6 *v* 9.0 to 5.0; *P* = 0.008). The relatively greater decline in frequency of depression among intervention participants was not statistically significant.
Gao L, 2012 [25]	China	Individual RCT	First-time pregnant women. Intervention group *n* = 96; control group *n* = 98	Standard care consisting of childbirth education	EPDS at 6 weeks and 3-months follow up		At 6 weeks postpartum: t = −4.05, *P* < 0.001; at 3-month postpartum: t = 2.39, *P* = 0.018.
Rahman A, 2008 [27]	Pakistan	Cluster RCT	Married women, third trimester of pregnancy with perinatal depression; 40 Union Council clusters. Intervention group *n* = 463; control group *n* = 440.	Enhanced usual care consisting of equal number of visits by untrained health worker	Infant weight and height at 12 months	Maternal depression using HDRS at 6 and 12 months	Mean difference at 6 months:−5·86; 95% CI −7·92 to −3·80; *P* < 0·0001. At 6 months: 78% reduction in prevalence of depression in intervention arm (AOR 0.22, 95% CI 0.14 to 0.36, *P* < 0.0001); At 12 months: 77% reduction (AOR 0.23, 95% CI 0.15 to 0.36, *P* < 0.0001).
Rojas G, 2007 [26]	Chile	Individual RCT	Mothers with major depression attending postnatal clinics with index children younger than 1 year. Intervention group *n* = 114; control group *n* = 116.	Usual care	Depressive symptoms using EPDS at 3 and 6 months after randomisation		Adjusted mean difference 3mo: −4.5 (−6.3 to −2.7), *P* < 0.0001 6mo: −2.3 (−0.50 to 0.04)
Tezel A, 2006 [28]	Turkey	A pre-test–post-test mutual controlled sem- experimental model.	Women all of whom had a risk of postpartum depression, but without exhibiting major depression symptoms. Intervention group *n* = 32; control group *n* = 30.	Nursing care	Depressive symptoms in postpartum period using the BDI after intervention		Significant difference in the prevalence of depressive symptoms before and after the intervention (McNemar test, *P* < 0.05). Both intervention and control (nursing care) groups showed significant reduction in mean scores from pretest to posttest (t = 10.062, *P* < 0.05 for control group and t = 5.462, *P* < 0.05 for intervention group).
Tripathy P, 2010 [29]	India	Cluster RCT	Open cohort of women 15–49 years who had just given birth from 36 clusters. Intervention group *n* = 6452; control group *n* = 5979.	Enhanced care with formation of cluster level committees.	Reduction in NMR and maternal depression score (K10) in year 2 and 3.	Secondary outcomes were stillbirths, maternal and perinatal deaths, uptake of antenatal and delivery services, home-care practices during and after delivery, and health-care-seeking behavior.	AOR: No or mild depression year 2: 0·91 (0·41–2·01) year 3: 2·33 (1·25–4·38); moderate depression year 2: 1·04 (0·50–2·16); year 3: 0·43 (0·23–0·80) Severe depression year 2: 1·53 (0·47–5·05) year 3: 0·70 (0·15–3·31) .

BDI, Beck's Depression Inventory; CES-D, Centre for Epidemiological Studies Depression Scale; CIS-R,: Revised Clinical Interview Schedule; EPDS, Edinburgh Postnatal Depression Scale; GHQ, General Health Questionnaire; HDRS, Hamilton Depression Rating Scale; K-10, Kesslers's 10-item scale; SCID, Structured Clinical Interviews for DSM IV Diagnoses; SF-36: Short Form 36; WHO-SRQ 20, World Health Organization Self-Reporting Questionnaire.

Depressive symptoms were measured using six different depression scales: the Edinburgh Postnatal Depression Scale (EPDS) (*n* = 3) ∗[21], ∗[25], ∗[26], [30], the Center for Epidemiological Studies Depression Scale (CES-D) (*n* = 2) ∗[22], ∗[24], Beck Depression Inventory (BDI) (*n* = 1) [28], the General Health Questionnaire (GHQ) (*n* = 1) [21], the Hamilton Depression Rating Scale (HDRS) (*n* = 1) [27] and the Kessler 10 (*n* = 1) [28]. Duration of the follow up ranged from 3 months to 3 years after treatment. All nine studies reported improvement in perinatal depression in the intervention compared with control groups (Table 1).

### Content of interventions

Content of interventions, and that of the adaptations (presented later), were extracted either from the study papers (*n* = 9) or from their linked papers (*n* = 3) [30], [31], [32]. In four studies, the interventions were adaptations of evidence-based psychological treatments; cognitive–behavioural therapy (CBT) (*n* = 2) ∗[24], ∗[27], interpersonal psychotherapy (IPT) (*n* = 1) and problem Solving therapy(*n* = 1) [28]. In one study, psychoeducation was adapted for relevance to postnatal care and delivered as part of a multicomponent stepped care intervention [26]. In another study, the intervention was an adaptation of an existing preventive mother–infant intervention programme [30], and, in three studies, the intervention was developed *de novo* for the study ∗[21], ∗[22], ∗[29]. The interventions as described in the studies were distilled into different strategies, and this has been presented in Table 2.

**Table 2:** Strategies distilled from various interventions included in the review.

Author	Intervention with theoretical basis (if any)	Child health education	Activating social networks	Psychoeducation	Psychostimulation	Cognitive restructuring	Problem solving	Behaviour activation	Befriending	Addressing interpersonal triggers
Aracena M, 2009 [21]	Home-visit programme	Yes	Yes	Yes	Yes					
Baker-Henningham, 2005 [22]	Early stimulation home visit programme	Yes	Yes		Yes				Yes	
Cooper PJ, 2009 [23]	Closely follows the principles contained in *The Social Baby*	Yes			Yes					
Futterman D, 2013 [24]	Cognitive–behavioural intervention plus peer-mentoring programme	Yes	Yes	Yes	Yes	Yes		Yes	Yes	
Gao L, 2012 [25]	Interpersonal psychotherapy-oriented childbirth education programme	Yes	Yes	Yes						Yes
Rahman A, 2008 [27]	Thinking healthy programme based on cognitive-behavioural therapy	Yes	Yes	Yes	Yes	Yes	Yes	Yes		
Rojas G, 2007 [26]	Psychoeducation as part of a multicomponent stepped-care intervention			Yes		Yes	Yes	Yes		
Tezel A, 2006[28]	Problem-solving training			Yes		Yes	Yes			
Tripathy P, 2010 [29]	Participatory women's group	Yes	Yes				Yes			

Most studies used interventions that consisted of various strategies targeting the mother, the mother–child dyad , the family or both. Strategies in which the mother was the main target were psychoeducation, cognitive restructuring, problem solving, behaviour activation, and befriending. Psychoeducation was the key component in one Chilean study [26], in which it was delivered as part of a multicomponent intervention that included structured pharmacotherapy if needed, systematic monitoring of clinical progress and treatment compliance, further training to doctors, and specialist supervision on a regular basis. Psychoeducation consisted of information about symptoms and the likely causes, offering hope and motivating women to seek appropriate treatment. This was also described in other studies where it was delivered either in the individual or group format ∗[21], ∗[24], ∗[25], ∗[27], ∗[28].

Cognitive restructuring was defined as becoming aware of one's thoughts to identify and label those which are helpful and unhelpful, and modify the unhelpful ones into more helpful ones, thereby improving symptoms of depression. Low-intensity cognitive restructuring using culturally appropriate pictures was used in the ‘Thinking Healthy Programme’ in Pakistan, where it incorporated the additional techniques of active listening, collaboration with family, guided discovery, and home work added to the routine practice of mother and child health education [27]. The Mamekhaya programme in South Africa too was adapted from CBT for relevance to prevention of mother-to-child transmission( PMTCT) services to focus on four broad topics: healthy living; feeling happy and strong; partnering and preventing transmission; and parenting [24].

Problem solving consisted of the five general stages: problem orientation, problem definition and formulation, generation of alternatives, decision making, and verification. In a Turkish study [28], training in problem solving was conceptualised as a form of self-control training; that is, the women ‘learns how to solve problems’ and thus discovers for herself the most effective way of responding [28]. Problem solving was used as an important strategy in the Thinking Healthy Programme, the Chilean psychoeducation intervention, and the participatory women's group intervention in India, where it addressed problems faced by mothers and their families ∗[26], ∗[27], ∗[28].

Behavioural activation (i.e. increasing behaviours that give the woman a sense of effectiveness and pleasure leading to improvements in thoughts and emotions) was used in three studies either as part of a cognitive behaviour intervention or independently ∗[24], ∗[26], ∗[27]. Non-specialist health workers, who were often mothers themselves, hence peers, performed the additional role of ‘befriending’, developing positive, supportive relationships with the depressed mothers, reducing their sense of isolation and providing individual assistance ∗[22], ∗[24].

Where the target was the mother–child dyad, the interventions focused on educating parents on the child's physical health, and also included healthcare practices for both mother and child, child nutrition and help seeking ∗[21], ∗[22], ∗[24], ∗[25], ∗[27], ∗[29], [30]. Apart from physical health, ‘psychostimulation’, defined as the provision of affection and warmth, responsiveness to the child, and the encouragement of autonomy and exploration, is an important aspect of perinatal care, and this is reflected in its use as the next common strategy across interventions ∗[21], ∗[22], ∗[24], ∗[27], [30]. Psychostimulation aimed to encourage the mother in sensitive, responsive interactions with her infant and thus sensitise the mother to her infant's individual capacities and needs. In one study [30], this was adapted from a preventive intervention programme by health visitors based on the principles contained in *The Social Baby* published by The Children's Project. This programme was adapted by incorporating the key principles of the World Health Organization's *Improving the psychosocial development of children*. Another study from Jamaica [22] developed an early stimulation home-visit programme, which focused on improving child development by improving mothers' knowledge and practices of child rearing and their parenting self-esteem. The NSHWs were trained to ensure that the mothers experienced success and feelings of competence. Some studies focused on discussion of parenting issues, including the importance of praise, attention, and responsiveness as well as appropriate discipline strategies.

Interventions that targeted the family and broader social milieu included strategies such as ‘activating social networks’ and ‘addressing interpersonal issues’. Activating social networks consisted of enlisting family and friends in various aspects of the intervention, including promoting adherence ∗[21], ∗[22], ∗[24], ∗[25], ∗[27], ∗[29]. It acknowledges the salience of social and family connectedness in many developing countries. A key component in a Chinese study was to address interpersonal (relationship) issues by understanding and dealing with emotional factors associated with these issues, especially where conflicts with husbands and mothers in law were frequently encountered [25].

### Adaptations to the interventions

Details of the cultural and contextual adaptations made to the interventions were categorised using Bernal's framework [20] and are presented in Table 3. Adaptations for language went beyond the literal translation to incorporate the use of colloquial expressions to replace technical terms, for example, using ‘stress’ instead of ‘depression’ and ‘thinking healthy’ instead of ‘cognitive– behaviour therapy’ [27]. Therapist adaptations, apart from using NSHWs most of whom were already available in the clinics and were often closely connected to local neighbourhoods, also used peers (i.e. mothers with experience in child rearing). These adaptations focused on therapist-patient matching to enhance the acceptability and credibility of the counsellor by emphasising shared experiences and awareness of local customs. The NSHWs attempted to develop friendly relationships with the mothers and to empathise with their expressed concerns ∗[22], ∗[26], ∗[27]. The use of metaphors to increase cultural relevance took the form of using material that was culturally appropriate; for example, a health calendar to monitor homework, the use of local stories and examples with characters resembling the patient's situation and background, and the use of idioms and symbols such as feeling cups to identify and quantify the intensity of feelings ∗[24], ∗[26], ∗[27], ∗[29]. These enabled the simplification of abstract concepts into more concrete, easy to understand terms. Cultural considerations were integrated into the content of the psychological treatment by focusing on pressing social concerns in the woman's life and addressing local customs; for example, issues related to Chinese postpartum practice ‘Zou Yue Zi’ ie. ‘doing the month’, which refers to the traditional Chinese custom of having new mothers rest for a month at home, often under the care of their mother-in-law [25]; and, in Pakistan, not expecting outdoor activities during the chilla (40-day confinement of mothers after delivery) when mothers do not go out of the house [27]. Adaptations in the dimension of concepts involved addressing cultural norms surrounding the concept of infancy and childcare practices, and focusing on relevant skill building techniques such as problem solving ∗[24], ∗[27], ∗[28]. Adaptations of goals involved development of client-derived treatment goals that were personally and culturally relevant, such as focusing on the health of the child and family unit rather than the mother. Goals were also extended beyond depression treatment; for example, by enhancing roles of self-help group members into community advocates and focusing on women's empowerment ∗[24], ∗[27]. Adaptations to methods such as reducing the focus on tasks requiring literacy (i.e. reading and writing) were important for ensuring applicability to low-literacy populations. Delivery of sessions at home or over the telephone and integrating with routine healthcare visits helped to increase acceptability and feasibility of intervention delivery as well as adherence ∗[25], ∗[26]. Adaptations to ensure that the psychological treatment fits into the patient's broader social context consisted of involving other family members in the intervention, focusing on interpersonal conflicts that may occur in joint family settings, and addressing issues related to the baby's gender (e.g. women attributed responsibility for the baby's gender to themselves especially in cultures that show preference for male children ∗[22], ∗[25], ∗[27]) (Table 3).

**Table 3:** Cultural adaptations described using Bernal's framework.

Principle	Adaptation	Rationale
Language: use of culturally centred language as part of the intervention		
Translation into local language	Manuals and patient materials were translated into the local language ∗[26], ∗[27].	To match the language spoken by patients and therapists to enhance understanding of the therapy concepts, methods and goals.
Technical terms replaced by colloquial expressions	Cognitive–behavioural therapy renamed ‘Thinking healthy’ Use of terms such as ‘stressed’ or ‘burdened’ where necessary and avoidance of psychiatric labels such as ‘depression’ [27].	Using literal translations or translations that are not culturally acceptable is one of the major barriers in therapy. Depression is not well understood as a term. Stressed or burden more understandable in the local context. To minimise stigma.
Therapist: consideration of the role of cultural similarities and differences in the client–therapist dyad		
Therapist –patient matching	Therapists matched from the same local community, speaking the local language ∗[26], ∗[27], ∗[29].	Local credibility and acceptability, fluency in local dialect, shared experience in norms and events impacting community, and familiarity with local idioms of distress.
Therapist–patient relationship	Therapist attempted to develop friendly relationships with the mothers and to empathise with their expressed concerns [22].	To ensure patient engagement in the treatment process.
Use of non-mental health workers	Use of Lady Health Worker within the primary care system, nurses, community workers. Role enhancement of the non-specialist health workers was highlighted most of whom were available in the clinics and were often closely connected to local neighbourhoods ∗[26], ∗[27].	To reduce stigma and preserve patient's privacy (especially during home visits) from inquisitive neighbours and family members. Also to make best use of already available, low cost resources.
Metaphors: the symbols and concepts that are shared by a particular cultural group		
Use of material with cultural relevance	Designation of a ‘health corner’ in each house, and a ‘health calendar’ provided to each mother to monitor homework and chart progress. Using culturally appropriate illustrations, for example, characters depicting mothers and infants [27].	By using the illustrated characters, the health workers could avoid direct confrontation with women and their families where it was not appropriate. It facilitated work with non-literate women.
Use of stories, local examples	Groups used methods such as picture-card games, role play, and story-telling to help discussions about the causes and effects of typical problems in mothers and infants, and devised strategies for prevention, homecare support and consultations. Case studies imparted through contextually appropriate stories [29].	Patient scould understand new ideas when described using familiar stories/figures and enhanced acceptability of treatment.
	Use of examples that were relevant to the specific population [26].	To increase cultural relevance
Use of idioms and symbols	Key domains were explored using tools such as: feeling cups to identify and quantify the intensity of feelings; the ‘feel, think, do’ method of problem solving and goal setting; and tokens to encourage peer support [24].	To convert an abstract concept such as mood into a more concrete, easy to understand concept.
Content: cultural uniqueness (values, customs) integrated into all aspects of the treatment		
Addressing stressors	Intervention to focus on addressing economic and social problems faced by mothers and families ∗[21], ∗[25].	Marked social problems interfere with recovery if left unaddressed.
Accounting for cultural norms surrounding the concept of infancy and child care practices	Focus on issues related to Chinese postpartum practice 'Zou Yue Zi' ie. ‘doing the month’, which refers to the traditional Chinese custom of having new mothers rest at home, often under the care of their mother-in-law, for a month after delivery [25].	To contextualise the treatment to address issues that are relevant to the cultural group.
	Ensuring culturally appropriate homework activities (e.g. not expecting outdoor activities during the chilla (40-day confinement of mothers after delivery) when mothers do not go out of the house [27].	To increase access to care and reduce participant burden. Acknowledgement of the traditions and values allowed the therapy teams entry into these families and increased the possibility of follow-through.
Concepts: the way in which the presenting problem of a woman is conceptualised and communicated		
Skill building	Problem solving was conceptualised as a form of self-control training, that is, the women ‘‘learns how to solve problems’ and thus discovers for herself the most effective way of responding [28].	To preserve congruence with cultural beliefs and physical/somatic belief models of illness causation.
	Cultural norms surrounding the concept of infancy and child care practices were taken into account with the aim of sensitising the mother to her infant's individual capacities and needs ∗[21], ∗[22], ∗[24].	
Goals: consideration of the specific values, customs, and tradition of the woman's culture when agreeing on treatment goals		
Client-derived goal	Focus on mother and infant health rather than maternal depression and have an a priori agenda of achieving optimal infant development through the intervention [27].	Infant care was seen as a shared responsibility and this helped engage not only the mother, but the whole family in a supportive role for the mother.
Extending goals beyond depression	Focus on empowerment - named the project Mamekhaya, which means ‘respect for women’ in Xhosa [24].	Addressing broader social issues for longer term impact.
	Emphasis was laid on group members' role development into community advocates as depression improves [27].	Underscoring impact of depression treatment on wider community development goals (e.g. farming initiatives, school attendance).
Methods: procedures followed for the achievement of the treatment goals		
Structural adaptations	Delivering treatment by telephone, home visits [25].	To increase accessibility and feasibility
	Integrating the intervention into routine day to day work of the non-specialist health workers [27].	
	Sessions arranged to follow routine childbirth education sessions with 20-min apart [25].	
Adaptation in techniques used to deliver treatment	Less use of written material and limiting homework to simple suggestions rather than writing tasks [26].	To overcome limited literacy levels.
	Worksheets for the mothers, with educational material related to the topics covered in the manual; personal diary, intended to provide the mother with a means through which she can reflect on her individual experience, share private thoughts and explore her own development [21].	To make treatment understandable and reinforce the therapists work.
Context: consideration of the woman's broader social, economic, and political context		
Context-specific issues addressed	Addressing issues related to baby's gender (e.g. women attributed responsibility for the baby's gender to themselves) [25].	Contextual stressors were seen as one of the major contributors to depression.
	Where other caregivers (for example, fathers, grandparents) were present, they were encouraged to take part in the intervention. Focus on improving relationship and reducing conflict with husbands as well as mothers in law ∗[22], ∗[25], ∗[27].	Acknowledges the central role of the family in the treatment process
	Home-made toys and books and materials in the home were used to keep the intervention low cost [22].	

### Intervention delivery

Issues related to intervention delivery are presented in Table 4, including the location, duration, format of intervention delivery, and the delivery agent. As described, most of the interventions for perinatal depression were integrated into existing health programmes, such as nutrition, child health, and development programmes, positive parenting programs, PMTCT programmes, routine childbirth education sessions, and community health programmes. Most of the interventions were delivered at home (*n* = 5) ∗[21], ∗[22], ∗[27], ∗[28], [30]. In three studies, the intervention was delivered in the clinic (though one study included an additional telephone session) ∗[24], ∗[25], ∗[26] and, in one study, the intervention was delivered in a community setting other than the woman's home [29]. Five studies delivered interventions in the individual format ∗[21], ∗[22], ∗[27], ∗[28], [30], whereas four used the group format ∗[24], ∗[25], ∗[26], ∗[29]. Number and frequency of sessions varied widely among the studies from three to 20 sessions delivered at weekly to monthly intervals over 6 weeks to 20 months.

**Table 4:** Details of intervention delivery including provider characteristics.

Author	Integration into mother and child health centre	Intervention delivery				Intervention provider				
		Location	Format	Duration	Treatment target -	Name	Qualification/s	Training duration	Supervision	NSHW characteristics/issues
Aracena M, 2009 [21]		Home	Individual	Average 12, hour-long sessions from third trimester to 1 year after delivery.	Mother and child	Health educators	Previous experience and they should haveraised children themselves.		By two nurse-midwives, 1 h per week.	
Baker-Henningham, 2005 [22]	Integrated into a nutrition and positive parenting programme.	Home	Individual	Weekly for half an hour over 1 year	Mother, child and other caregivers when available	Community health aides	Para-professionals employed in government health centres.	6 weeks; 4 weeks training on health and nutrition and 2 weeks training in child development, parenting issues.	The supervisor observed each aide conducting visits once a month and visited the health centre every fortnight to discuss the programme and review the records of each visit.	
Cooper PJ, 2009 [23]	Integrated into a child development programme.	Home	Individual	16 sessions starting antenatally at weekly, fortnightly and monthly intervals ending at 5 months Postpartum.	Mother-infant relationship	Lay community health workers	No formal specialist qualifications; all were mothers selected in consultation with the local community council.	4-month training in basic parenting and counselling skills and the specific mother-infant intervention.	An experienced community clinical psychologist provided session by session supervision in the group format, supervision, weekly.	NSHW had a focused task(rather than responsibility for comprehensive community health) and had strong community support.
Futterman D, 2013 [24]	Integrated into a PMTCT program for HIV positive women.	Clinic	Group	Eight sessions	Mother	Mothers; two mothers - mentor mothers	Mentor mothers who were alsoHIV-positive, had a child recently, had used PMTCTservices, and were coping positively.			
Gao L, 2012 [25]	Integrated with routine childbirth education sessions.	Two clinic based and one telephone session.	Group	Three sessions: Two 90-min antenatal group sessions; one telephone follow up session within 2 weeks of delivery.	Mother	Midwife educator		Intensive training and supervision (not described).		
Rahman A, 2008 [27]	Integrated into a community health programme.	Home	Individual	16 sessions from the last month of pregnancy until 10 months postpartum.	Focus on mother and infant health	Lady health worker	Mostly high-school completers	2-day training workshop and 1-day refresher after 4 months.	Monthly supervision by mental health specialists in group format for half a day. Emphasis on experiential learning through shared experiences of the group.	NSHW were from the same community as patients, and understand the socio-cultural context of their problems. Their existing job included visiting household and talking to the family about primary prevention. Many were trusted ‘health educators' within their community and thus are able to adopt the CBT therapist's role and access the families with relative ease.
Rojas G, 2007 [26]		Clinic	Group	Groups consisted of 1 session per week for 8 weeks, each session lasting 50 mins.		Midwives or nurses		8 h	Weekly, by a designated trained, non-professional person who monitored attendance at consultations and groupsessions and provided support and advice.	
Tezel A , 2006 [28]		Home	Individual	6 weekly sessions	Mother	Nurse researcher			Study was part of doctoral thesis of the nurse researcher and was supervised. No details.	Nurse had two professional roles: that of a caregiver and an educator.
Tripathy P, 2010 [29]		Community	Group	20 monthly meetings	Mother and child		A local woman, selected on the basis of criteria (including speaking the local language and having the ability to travel to meetings) identified by the community.	a 7-day residential training course	Fortnightly meetings with district co-ordinators.	

NSHW, non-specialist health workers; PMTCT, prevention of mother-to-child transmission.

To ensure that the intervention is provided competently and that it can be generalised to other situations and settings, the nature of the intervention needs to be clearly described and documented and the accuracy of implementation verified. All the studies incorporated mechanisms to promote fidelity, but these varied in form and application. Five mechanisms were used to maximise fidelity in the studies reviewed: use of a manualised intervention; attention to NSHW recruitment; NSHW training; regular supervision; and assessment of therapy quality. All the interventions were structured, involving specific content and a prescribed number of sessions or duration of programme. In three interventions, these details were documented in a programme-specific manual ∗[21], ∗[23], ∗[27]. The remaining studies do not specifically mention the use of an intervention manual.

Five studies used paraprofessionals already working within the health system, such as community health workers, midwives and nurses, three studies recruited women who were mothers themselves ∗[21], ∗[23], ∗[24], and hence could be considered peers, and one study used lay women from the community without specifying they were mothers ∗[21], ∗[23], ∗[24], ∗[29]. Two studies described the characteristics of the NSHW: these were women from the local community who had no formal training, apart from that received from the study team for delivery of the intervention. In addition, they had a focused task (rather than responsibility for comprehensive community health), they were given appropriate support and supervision, and they had strong community support, all of which are regarded as essential for effective community health worker programmes. Furthermore, they were selected in consultation with the local community council [23]. The NSHW understood the sociocultural context of the women's problems and, as many were trusted ‘health educators’ within their community, they were able to take on the therapist's role and access the families with relative ease [27].

Non-specialist health worker training varied in length from 12 hours [26] to 4 months [23]. All studies report supervision of NSHW by specialists throughout the duration of the study, ranging in frequency and intensity from weekly ∗[21], ∗[23], ∗[26] to monthly supervision [27], either in the individual or group format. The effectiveness of the intervention depends on the therapy quality and competence of the counselor [33], which can be assessed by various means: the evaluation of patient outcomes (e.g. symptom reduction); the assessment of individual sessions; and evaluation of standardised role plays. None of the studies describe assessment of NSHW competence to confirm acquisition of knowledge or skills. Only one study reported assessing therapy quality [23]. In this study, written records were reviewed weekly as part of group supervision. In addition, during the pilot, the NSHW tape-recorded all sessions of their last two cases, and a random selection of these were transcribed and subjected to content analysis. The transcripts were coded by an independent rater to quantify the presence of essential counselling skills and strategies required by the programme. On the basis of ordinal ratings of global categories, the study reports that all NSHW showed at least moderate to good performance, with two of the four NSHW being rated as excellent on all dimensions, confirming therapy quality was satisfactory.

### Challenges encountered

Numerous practical and cultural barriers were experienced in the delivery of the interventions. Common practical barriers were poor adherence, economic cost of home visits, and lack of private space for delivery of the interventions. Increased work pressure and low motivation of NSHW was also reported. The cultural barriers consisted chiefly of low acceptability of ‘talking treatment’, stigma of mental health interventions, and salience of social problems that demand appropriate attention over and above the counselling interventions. Most of these challenges were addressed by the various adaptations described above, although some challenges remain unaddressed. For example, although the use of NSHW is a potentially low-cost strategy to increase the coverage of evidence based care in LMIC, a limitation of this approach is its sustainability and feasibility when taken to scale. Continuous work with depressed and psychosocially deprived women could lead to burnout or a drop in efficiency of NSHW already carrying a number of other responsibilities. It is also important to ensure optimum quality of the intervention being delivered. If such programmes were to be implemented at a larger scale, it would be necessary to have scalable training, supervision and monitoring mechanisms to ensure NSHW competence and treatment quality. The lack of assessment methods in all but one of the reviewed studies is a major challenge in determining NSHW competence and quality of intervention delivery. The other potential challenge is the utility of this approach in settings where there are no NSHWs within the health system. In such circumstances, it is important to ensure the intervention is simple enough to be taught to volunteer peer-workers and family members. The interventions can be further adapted for telephone delivery and, with literate women, it may even be possible to have self-guided versions of the intervention. This, in addition to reducing costs, could be important in enhancing treatment adherence.

## Conclusion

In this review, we included nine studies demonstrating that NSHW delivered psychosocial interventions for perinatal depression are feasible in LMIC. The interventions incorporated important features that had particular relevance to LMIC. These are community-based delivery provided within the context of maternal and child health beginning in the antenatal period and extending postnatally, focus of the intervention beyond the mother to include the child and involving other family members, attention to social problems such as domestic violence, substance abuse and HIV, and a focus on empowerment of women. The interventions had active psychotherapeutic components derived from CBT and interpersonal therapy, in addition to general supportive measures, such as empathic listening. The key components of the interventions were two-fold: information giving and skill building. The information-giving components included parenting skills, basic healthcare practices, and information about perinatal depression and care-seeking, whereas the skill-building components included communication skills, behavioural activation, and problem solving. All the interventions were adapted for contextual and cultural relevance, and to be deliverable by NSHW. Delivery of the interventions was aided by keeping them simple and structured, for example, the low intensity cognitive restructuring delivered as part of the ‘Thinking healthy program’. Ensuring adequate training of NSHW was important but not sufficient in itself to ensure optimum intervention delivery. The training was, in every instance, followed up with structured supervision either provided in the individual or group format.

**Practice points**

This review demonstrates the feasibility of task shifting using NSHW to deliver psychological interventions for perinatal depression in low-resource settings where specialist services are both scarce and expensive. Key features that distinguish these interventions from those typically offered in specialist settings include:

• use of health workers who are embedded in their clients' context and are performing general maternal health care roles;
• delivery in settings that are closer to the mother (e.g. her own home);
• use of language, metaphors and content that are culturally appropriate;
• using a range of strategies addressing informational needs and skills; and
• addressing the mother's primary concerns, such as their child's wellbeing or social problems, alongside their mental health distress.

**Research agenda**

• Cost-effectiveness studies of perinatal mental health interventions.
• Evaluation of collaborative care interventions, where specialist care is integrated with NSHW care, for the full range of perinatal mental health problems.
• Evaluation of interventions for perinatal mental health problems led by a peer support worker.
• Studies documenting long-term benefits of perinatal mental-health interventions on child health and development.
• Evaluation of the effect of scaled-up programmes for perinatal mental health care.
• Measures to evaluate counsellor competence and quality of scaled-up perinatal mental health programmes.

## Appendix 1 Search terms used for Medline and EMBASE.

1. exp depressive disorder/
2. exp depression/
3. depress$.tw.
4. maternal depression.mp. or Depression, Postpartum/
5. or/1-4 [DEPRESSION]
6. Allied Health Personnel/
7. Community Health Aides/
8. Nurses Aides/
9. Psychiatric Aides/
10. Caregivers/
11. Voluntary Workers/
12. Community Networks/
13. exp Self-Help Groups/
14. Social Support/
15. Health Manpower/
16. (Personnel Staffing and Scheduling).mp. [mp = title, abstract, original title, name of substance word, subject heading word, keyword heading word, protocol supplementary concept, rare disease supplementary concept, unique identifier]
17. (lay adj3 (worker? or visitor? or attendant? or aide or aides or support* or person* or helper? or carer? or caregiver? or care giver?)).mp.
18. ((voluntary or volunteer?) adj3 (worker? or visitor? or attendant? or aide or aides or support* or person* or helper? or carer? or caregiver? or care giver? or consultant? or advisor? or counselor? or counsellor? or assistant? or staff)).mp.
19. (untrained adj3 (worker? or visitor? or attendant? or aide or aides or support* or person* or helper? or carer? or caregiver? or care giver? or consultant? or advisor? or counselor? or counsellor? or assistant? or staff or nurse? or doctor? or physician? or therapist?)).tw.
20. (trained adj3 (worker? or visitor? or attendant? or aide or aides or support* or person* or helper? or carer? or caregiver? or care giver? or consultant? or advisor? or counselor? or counsellor? or assistant? or staff or nurse? or doctor? or physician? or therapist? )).tw.
21. (unlicensed adj3 (worker? or visitor? or attendant? or aide or aides or support* or person* or helper? or carer? or caregiver? or care giver? or consultant? or advisor? or counselor? or counsellor? or assistant? or staff or nurse? or doctor? or physician? or therapist?)).tw.
22. ((nonprofessional? or non professional?) adj3 (worker? or visitor? or attendant? or aide or aides or support* or person* or helper? or carer? or caregiver? or care giver? or consultant? or advisor? or counselor? or counsellor? or assistant? or staff )).tw.
23. ((non medical or non health or non healthcare or non health care) adj3 (worker? or visitor? or attendant? or aide or aides or support* or person* or helper? or carer? or caregiver? or care giver? or consultant? or advisor? or counselor? or counsellor? or assistant? or staff )).tw.
24. (community adj3 (worker? or visitor? or attendant? or aide or aides or support* or person* or helper? or carer? or caregiver? Or care giver? or consultant? or advisor? or counselor? or counsellor? or assistant? or staff )).tw.
25. (paraprofessional? or paramedic or paramedics or paramedical worker? or paramedical personnel or allied health personnel or allied health worker? or support worker? or non specialist? or specially trained or barefoot doctor? or nurse* aide? or psychiatricaide? or psychiatric attendant? or social worker? or teacher? or school staff or trainer?).tw.
26. (health* adj3 (auxiliary or auxiliaries)).tw.
27. (nurs* adj1 (auxiliary or auxiliaries)).tw.
28. (informal adj (caregiver? or care giver? or carer?)).tw.
29. (self help group? or support group?).tw.
30. ((social or psychosocial) adj (care or support)).tw.
31. (village adj3 worker?).tw.
32. community based.tw.
33. (community adj3 intervention?).tw.
34. community network?.tw.
35. ((health or health care or healthcare) adj manpower).tw.
36. human resources.tw.
37. (task? adj3 shift*).tw.
38. (staff* adj3 chang*).tw.
39. or/6-38 [NSHW ]
40. exp Developing Countries/
41. (Afghanistan or Kyrgyz Republic or Bangladesh or Liberia or Benin or Madagascar or Burkina Faso or Malawi or Burundi or Mali or Cambodia or Mauritania or Central African Republic or Mozambique or Chad or Myanmar or Comoros or Nepal or Congo, or Niger or Eritrea or Rwanda or Ethiopia or Sierra Leone or Gambia or The Somalia or Guinea or Tajikistan or Guinea-Bissau or Tanzania or Haiti or Togo or Kenya or Uganda or Korea, Zimbabwe). hw,kf,ti,ab,cp.
42. (((Algeria or Libya or American Samoa or Lithuania or Angola or Macedonia or Antigua Barbuda or Malaysia or Argentina or Maldives or Azerbaijan or Mauritius or Belarus or Mexico or Bosnia) and Herzegovina) or Montenegro or Botswana or Namibia or Brazil or Palau or Bulgaria or Panama or Chile or Peru or China or Romania or Colombia or Russian Federation or Costa Rica or Serbia or Cuba or Seychelles or Dominica or South Africa or Dominican Republic or St Lucia or Ecuador or St Vincent or The Grenadines or Gabon or Suriname or Grenada or Thailand or Iran Islamic Republic or Iran or Tunisia or Jamaica or Turkey or Jordan or Turkmenistan or Kazakhstan or Tuvalu or Latvia or Uruguay or Lebanon or Venezuela).hw,kf,ti,ab,cp.
43. (((((Albania or Moldova or Armenia or Mongolia or Belize or Morocco or Bhutan or Nicaragua or Bolivia or Nigeria or Cameroon or Pakistan or Cape Verde or Papua New Guinea or Congo Republic or Paraguay or Cote d Ivoire or Philippines or Djibouti or Samoa or Egypt Arab Republic or Egypt or Sao Tome) and Principe) or El Salvador or Senegal or Fiji or Solomon Islands or Georgia or South Sudan or Ghana or Sri Lanka or Guatemala or Sudan or Guyana or Swaziland or Honduras or Syrian Arab Republic or India or Timor Leste or Indonesia or Tonga or Iraq or Ukraine or Kiribati or Uzbekistan or Kosovo or Vanuatu or Lao PDR or Vietnam or Lesotho or West Bank) and Gaza) or Marshall Islands or Yemen Republic or Micronesia Federal States or Zambia).hw,kf,ti,ab,cp.
44. ((developing or less* developed or under developed or underdeveloped or middle income or low* income or underserved or under served or deprived or poor*) adj (countr* or nation? or population? or world)).ti,ab.
45. ((developing or less* developed or under developed or underdeveloped or middle income or low* income) adj (economy or economies)).ti,ab.
46. (low* adj (gdp or gnp or gross domestic or gross national)).ti,ab.
47. (low adj3 middle adj3 countr*).ti,ab.
48. (lmic or lmics or third world or lami countr*).ti,ab.
49. transitional countr*.ti,ab.
50. or/40-49 [LMIC]
